# Perinatal Risk Factors and Clinical Correlations in Molar–Incisor Hypomineralization: A Cross-Sectional Epidemiological Study

**DOI:** 10.3390/epidemiologia7010004

**Published:** 2025-12-26

**Authors:** Esztella-Éva Kis, Ilona-Boglárka Gecse, Cristina Bica, Csaba Dudás, Henrietta Dudás, Krisztina Martha

**Affiliations:** Faculty of Dental Medicine, George Emil Palade University of Medicine, Pharmacy, Science and Technology of Târgu Mures, 38 Gh. Marinescu Str., 540139 Târgu Mureș, Romania; esztella.kis@umfst.ro (E.-É.K.); bgecse@yahoo.com (I.-B.G.); csaba.dudas@umfst.ro (C.D.); maria-henrietta.dudas@umfst.ro (H.D.); krisztina.martha@umfst.ro (K.M.)

**Keywords:** molar–incisor hypomineralization, enamel defects, pediatric dental epidemiology, pregnancy

## Abstract

Background: Molar–Incisor Hypomineralization (MIH) represents a developmental enamel defect of systemic origin, typically affecting the first permanent molars and often the incisors. Within the limitations of this study, several associations were observed between perinatal factors and MIH-related outcomes. However, most of these connections were not retained in adjusted analyses. Febrile illness during the first year of life showed a significant association with hypersensitivity. Methods: A structured 30-item questionnaire was distributed to mothers of 50 children diagnosed with MIH between February and March 2024. Data was analyzed using chi-square tests, with *p* < 0.05 considered significant, and univariate and multivariate logistic regressions at 95% confidence interval. Clinical diagnosis followed the Weerheijm (EAPD) criteria. Results: Maternal medication during pregnancy (antibiotics, antiepileptics, asthma drugs) was significantly associated with preterm birth (*p* = 0.01). Low birth weight correlated with tooth eruption disorders (*p* = 0.009) and perinatal complications such as hypoxia and respiratory distress (*p* = 0.0001). Fluoride application demonstrated a protective effect against discolorations (*p* = 0.005), caries (*p* = 0.002), and hypersensitivity (*p* = 0.01). In the multivariate model, febrile illness during the first year of life may be associated with hypersensitivity in MIH-affected teeth (aOR = 5.71, 95% CI: 1.01–32.27, *p* = 0.049). Conclusions: Maternal medication and perinatal complications, particularly low birth weight, were associated with MIH occurrence. Preventive strategies emphasizing maternal health, early screening, and remineralization-based therapies can mitigate long-term oral health impacts.

## 1. Introduction

MIH is a qualitative enamel defect characterized by demarcated opacities, post-eruptive breakdown, and increased tooth sensitivity. It affects approximately 10–25% of children worldwide, representing a growing public health concern [1,2]. Over the past two decades, epidemiological investigations into developmental enamel defects have increasingly focused on MIH and related conditions.

Diagnostic approaches were largely consistent, with the EAPD (European Academy of Paediatric Dentistry) criteria most frequently employed, and longitudinal analyses indicate that global MIH prevalence has remained relatively stable over the past two decades [3]. EAPD clinical guidance highlights systemic insults during enamel formation as candidate risk factors for MIH. We therefore targeted prenatal medication categories that biological plausibility or prior epidemiology has implicated (antibiotics, antiepileptics, asthma medications) while collecting alcohol, tobacco, and illicit drug use as distinct variables. Clinically, MIH leads to structural weakness, hypersensitivity, and caries susceptibility, often requiring repeated dental interventions [4].

The etiology of MIH is complex, with evidence pointing toward systemic disturbances during the mineralization phase of amelogenesis [5].

Numerous studies have explored possible prenatal, perinatal, and postnatal systemic factors associated with molar–incisor hypomineralization, including maternal medication use, pregnancy complications, prematurity, low birth weight, and early childhood infections. Although several associations have been proposed, the available evidence remains inconclusive, and results are often inconsistent across populations. Differences in exposure measurement, recall-based data, and diagnostic criteria further complicate interpretation. Importantly, there is limited evidence linking perinatal risk factors to specific clinical characteristics of MIH, such as hypersensitivity or eruption disturbances. Addressing these gaps requires additional population-based studies that evaluate systemic risk factors in relation to both the occurrence and clinical expression of MIH [6,7,8]. Perinatal and early life stressors can disrupt ameloblast activity, leading to defective enamel mineralization. From a public health perspective, understanding these risk factors is essential for designing maternal care and pediatric prevention programs.

This study was conducted among children from Târgu Mureș, Romania, a region for which data on molar–incisor hypomineralization remain limited, despite documented variability in MIH prevalence and risk factors across Europe. The availability of detailed perinatal histories and standardized clinical assessments in this pediatric population provided a valuable opportunity to explore systemic determinants of MIH within a defined regional context. We hypothesized that exposure to adverse prenatal, perinatal, and early-life systemic factors—particularly prematurity, low birth weight, maternal medication use, and early febrile illness—would be associated with the occurrence and clinical expression of MIH, including hypersensitivity and enamel-related disturbances. Accordingly, this study aimed to evaluate perinatal risk factors and their clinical correlations in children diagnosed with MIH.

## 2. Materials and Methods

Ethical approval was obtained from the George Emil Palade University Institutional Review Board (IRB approval number: 2822/16.02.2024). Written informed consent was obtained from all parents or guardians before participation. The structured 30-item questionnaire underwent face and content validation by two pediatric dental specialists; items were refined for clarity before data collection. Participants were recruited consecutively from three pediatric dental clinics in Târgu Mureș, Romania, between February and March 2024. Diagnosis of MIH followed the Weerheijm criteria (European Academy of Peadiatric Dentistry adaptation): presence of demarcated opacities, post-eruptive enamel breakdown, atypical restorations, or extraction due to MIH, affecting at least one first permanent molar and/or incisors.

The questionnaire was developed from previously published instruments and refined by two independent pediatric dentists for content validity. It was piloted with five parents to ensure clarity and comprehension before data collection. Variables included maternal medication during pregnancy (antibiotics, chemotherapeutics, asthma medication, antiepileptics), birth complications (hypoxia, prematurity, low birth weight), and early childhood diseases (pneumonia, tonsillitis, gastrointestinal or urinary infections). Systemic diseases such as diabetes, allergies, cardiovascular diseases, epilepsy, and hypocalcemia were also recorded (see Supplementary Table S1).

Outliers in continuous variables were evaluated using a Grubbs’ test for normally distributed data (α = 0.05). To investigate factors associated with hypersensitivity in children with MIH, univariate and multivariate logistic regression analyses were performed using IBM SPSS Statistics version 25 (IBM Corp., Armonk, NY, USA). Predictor variables included maternal medication use, low birth weight (<2500 g), preterm birth (<37 weeks), fluoride exposure, and febrile illness during the first year of life. Associations are presented as crude (OR) and adjusted odds ratios (aOR) with 95% confidence intervals (CI) and corresponding *p*-values.

A post hoc power analysis was conducted based on the total sample size of 50 participants using G*Power 3.1.9.7 software (Heinrich-Heine-Universität, Düsseldorf, Germany). At a significance level of 0.05, the analysis had an estimated statistical power of approximately 94% to detect a medium effect size and 100% for a large effect size, while power was lower (29%) for detecting small effects.

## 3. Results

The mean age of children was 8.15 ± 1.25 years (Table 1), corresponding to the optimal age for MIH diagnosis, as all first permanent molars and most incisors are erupted. The gender distribution included 21 boys (42%) and 29 girls (58%).

Sixteen percent of the interviewed mothers had some kind of chronic illness, 26% of them declared smoking or alcohol consumption during pregnancy, and 12% of the mothers had antibiotic therapy during the first half of the pregnancy. Twenty-six mothers out of 50 had C-section, and only 29% of the births were without any complications. Eight percent of the respondents declared hypoxia, 33% premature birth, 9% breathing difficulties of the infant right after birth, and 21% other complications during birth. Regarding birth weight, 24 babies out of 50 had weight under 2500 g (Table 2). Thirty-two percent of the children have some kind of allergy (all of them external type), 58% of them had antibiotic treatment before the age of 1 year, and in 12% there was no breastfeeding attempt at all. Twenty percent of the MIH patients have some kind of systemic disease; in 14% of the cases, this was diagnosed before the age of 1 year, and in 18% of these cases, long-term medication has been prescribed. Eighteen percent of the examined children undergo systemic fluoride prophylaxis, 60% of them use fluoride toothpaste, and 62% of them reveal enamel lesions or discolorations.

Because the sample size was insufficient to support sex-stratified hypothesis testing, analyses were conducted using the combined dataset only. The analysis revealed several significant associations between perinatal factors and MIH-related outcomes (Table 3). Maternal medication during pregnancy was significantly linked with preterm birth (*p* = 0.01). Low birth weight correlated with delayed eruption (*p* = 0.009) and birth complications such as hypoxia and respiratory distress (*p* = 0.0001). Fluoride application showed a strong protective effect against enamel discoloration, caries, and hypersensitivity (*p* = 0.005, *p* = 0.002, *p* = 0.01, respectively). No significant association was found between breastfeeding, delivery mode, or febrile illness in the first year of life and MIH prevalence.

In univariate analyses, none of the examined factors were found to be statistically significant (*p* > 0.05) (Table 4). However, in the multivariate model, febrile illness during the first year of life was identified as a significant predictor of hypersensitivity in children with MIH (aOR = 5.71, 95% CI: 1.01–32.27, *p* = 0.049). No significant associations were observed for maternal medication use, low birth weight, preterm birth, or fluoride exposure in the adjusted analysis (*p* > 0.05).

## 4. Discussion

This exploratory study investigated the associations between perinatal and early-life systemic factors and MIH in a pediatric population. In univariate analyses, febrile illness during the first year of life was not significantly associated with hypersensitivity in MIH-affected teeth; however, after multivariate adjustment, it emerged as a significant factor (aOR: 5.71, 95% CI: 1.01–32.27, *p* = 0.049). Other tested factors—including maternal medication, low birth weight, preterm birth, and fluoride exposure—showed trends toward association but did not reach statistical significance after adjustment. These findings highlight the potential role of early systemic influences on enamel development while underscoring the exploratory nature of the study. The enamel mineralization process is susceptible to systemic stress, especially during the late gestational and early postnatal periods, when ameloblasts undergo final differentiation [9]. Antibiotic and antiepileptic exposure during pregnancy may alter calcium homeostasis and mitochondrial function, impairing enamel formation [10,11]. A recent systematic review identified 138 cross-sectional studies encompassing nearly 200,000 children and adolescents aged 3 to 18 years across 53 countries, reporting highly variable MIH prevalence ranging from 0.5 to 0.6% in Poland to 46.6% in Brazil, reflecting substantial geographical and population heterogeneity [3]. Global pooled meta-analytic estimates indicated a prevalence of 25.3% for enamel hypomineralisation, 15.5% for MIH, and 6.9% for combined molar–incisor hypomineralisation, with North America showing the highest prevalence (23.9%) and Africa the lowest (12.8%) [3,12]. In Europe, prevalence varies between 11.5% and 18.6%, while Spain reports higher rates ranging from 12% to 28.6% [12,13]. In Madrid specifically, a study using EAPD criteria found a prevalence of 28.63% among children aged 8–16 years, comparable to findings from Caracas, Venezuela (25.35%), Brazil (28.7%), Lebanon (26.7%), and Mexico (12.3–35.4%) [13,14].

The adjusted association between febrile illness in the first year of life and hypersensitivity in MIH-affected teeth is consistent with prior evidence suggesting systemic stress during early childhood may disrupt ameloblast function and enamel mineralization. Supporting studies have reported similar links between recurrent early-life fever and enamel hypomineralization, whereas other investigations have failed to detect a significant association, possibly due to differences in study design, population, or definitions of febrile episodes. Biologically, febrile illness may provoke systemic inflammatory responses that interfere with enamel prism organization, increasing porosity and susceptibility to hypersensitivity. The observed effect aligns with prior reports of increased enamel fragility following metabolic or systemic insults during the critical postnatal maturation period [15,16].

Several univariate associations were observed, including low birth weight with tooth eruption disorders and perinatal complications (hypoxia, respiratory distress), as well as fluoride exposure with enamel discoloration and caries occurrence. These findings are supported by previous regional studies and mechanistic research, which suggest that compromised mineralization or altered calcium homeostasis may underlie enamel defects. Our findings are consistent with a similar study from our region showing that early-life factors can influence enamel development. Similar to the regional study, we observed that low birth weight was significantly associated with tooth eruption disorders and perinatal complications such as hypoxia and respiratory distress. Additionally, fluoride application correlated with both enamel discoloration and caries occurrence, demonstrating the impact of postnatal exposures on dental outcomes. While the other study reported that prolonged labor, perinatal and early postnatal medication, and higher maternal age increased the risk of MIH, our results similarly emphasize the role of early-life biological and environmental factors in dental development. Together, these findings emphasize the multifactorial etiology of enamel defects, involving both prenatal/perinatal conditions and postnatal interventions, and support the need for careful monitoring and preventive strategies in at-risk children [17]. Although some differences were not statistically significant in our study, these trends support the multifactorial etiology of MIH and its potential co-occurrence with other dental anomalies. However, a meta-analysis from 2021 indicated no significant sex-related difference in MIH prevalence (OR = 0.986, 95% CI 0.940–1.035, I^2^ = 32.6%, *p* = 0.564), reporting an overall prevalence of 13.5%, with 36.3% of cases classified as moderate to severe, 36.6% involving incisors, 3.6% presenting hypomineralization of second primary molars, and significant associations with slightly older age, maternal age, prolonged labor, perinatal and early postnatal medication, and hypodontia [12]. In contrast, a recent regional study from Madrid found that MIH was more prevalent among girls (85; 60.71%) than boys (55; 39.28%), whereas other studies have reported a male predominance, highlighting the variability of sex-related findings at the individual study level [13]. Further evidence supports the multifactorial etiology of MIH. Children who experience health complications during early childhood, as well as those whose mothers encountered illnesses during pregnancy, are at increased risk [18]. The structural fragility and increased porosity of affected enamel elevate susceptibility to dental caries and hypersensitivity, with inflammatory responses in the pulp altering sensory neuron activity; consequently, even under local anesthesia, affected teeth may respond to cold or painful stimuli, influencing pediatric patient behavior during treatment [18]. A recent case–control study (2025) identified vaginal delivery and a history of varicella as protective factors, whereas preterm birth, frequent analgesic use, and recurrent diarrhea were modestly associated with MIH, while no clear links were observed with vitamin D deficiency, short-term breastfeeding, hypoxia at birth, or high fever, highlighting the complex interplay of prenatal, perinatal, and postnatal factors [12]. Additionally, meta-analyses of large cohorts demonstrated moderate to high increased risks associated with epigenetic influences (monozygotic twins), genetic variants (SNPs), maternal medication during pregnancy, and vitamin D deficiency [4]. Fluoride exposure demonstrated a strong protective association, reinforcing prior findings that the use of appropriate fluoride varnish and remineralizing treatments (e.g., CPP-ACP formulations) enhances enamel resistance to demineralization [3,19,20]. These findings emphasize the need for preventive measures targeting both maternal health and early pediatric dental care. Public health implications are considerable: MIH not only affects dental esthetics but also quality of life, due to pain, hypersensitivity, and repeated treatments [17,21]. In this study, febrile illness during the first year of life was found to be associated with hypersensitivity in children with MIH (aOR: 5.71, 95% CI: 1.01–32.27, *p* = 0.049). This observation supports the hypothesis that early-life systemic influences may contribute to the pathophysiology of hypersensitivity in MIH-affected teeth. The prevalence of dentin hypersensitivity and toothache in MIH-affected patients has been estimated at 45% per patient, and 22% per tooth [22]. The intensity of hypersensitivity was found to be higher in MIH molars compared to non-MIH teeth, with logistic regression showing molar teeth (OR 5.49, 95% CI 1.42–21.27) and teeth with enamel breakdown (OR 4.61, 95% CI 1.68–12.63) to be especially affected [23]. Although local anesthesia provides sufficient pain control during treatment, it does not fully eliminate sensitivity to cold or discomfort, suggesting that alternative management strategies and further histopathological studies are needed to address hypersensitivity in MIH-affected teeth [24]. Children with MIH often develop dental anxiety, which can persist into adulthood. Integrating MIH screening into national dental check-up programs could enable early intervention and reduce long-term costs [25,26]. Further, the inclusion of maternal education regarding safe medication use and adequate prenatal care is essential to mitigate MIH incidence. Clinically, MIH-affected teeth present treatment challenges. Their porous enamel compromises bonding efficacy, increasing restoration failure rates. Minimally invasive techniques, such as resin infiltration (ICON), remineralization, and fluoride varnish applications, should be prioritized. Recent studies have shown that pre-treatment remineralization followed by infiltration significantly improves esthetic and structural outcomes [27,28,29,30]. This approach aligns with preventive, patient-centered public health strategies.

This study has several limitations. The relatively small sample size limits statistical power and precision of effect estimates. The cross-sectional design precludes causal inferences, and reliance on parental recall introduces potential reporting bias. Some variables, including timing and severity of febrile illness or medication exposure, were imprecisely measured, which may attenuate observed associations. Despite these limitations, the study provides valuable preliminary insights into the multifactorial etiology of MIH and identifies early-life systemic factors for further investigation. These exploratory findings suggest potential trends that need confirmation in larger studies.

In summary, febrile illness during the first year of life may be associated with hypersensitivity in MIH-affected teeth, highlighting the potential impact of early systemic stress on enamel development. Other perinatal and postnatal factors showed suggestive trends but were not significant after adjustment. These findings support the multifactorial nature of MIH and emphasize the need for larger, prospective studies to clarify causal pathways and guide preventive strategies. Clinically, identifying children at risk may inform early monitoring and minimally invasive interventions to mitigate the consequences of MIH.

## 5. Conclusions

This study found a statistically significant association between febrile illness during the first year of life and hypersensitivity in MIH-affected teeth, based on adjusted analyses. Other associations observed in this study should be interpreted cautiously due to the small sample size and study limitations. Future research using larger, longitudinal designs is warranted to confirm these findings.

## Figures and Tables

**Table 1:** Characteristics of study participants.

Characteristics	n (%)
Age	
Age (years), mean ± SD	8.15 ± 1.25
Sex	
Male	21 (42%)
Female	29 (58%)
Perinatal factors	
Low birth weight (<2500 g)	22 (44%)
Preterm birth (<37 weeks)	17 (34%)
Maternal medication during pregnancy	13 (26%)
Early-life exposures	
Fluoride exposure	30 (60%)
Febrile illness < 1 year	15 (30%)

**Table 2:** Distribution of perinatal and early-life factors by sex.

Variable	Male n (%)	Female n (%)	Fischer’s Exact Test	Total n
Maternal medication	6 (46%)	7 (54%)	0.75	13
Low birth weight	11 (50%)	11 (50%)	0.39	22
Preterm birth	8 (47%)	9 (53%)	0.76	17
Fluoride exposure	13 (43%)	17 (57%)	1.00	30
Febrile illness < 1 year	8 (53%)	7 (47%)	0.36	15

**Table 3:** Significant and non-significant associations between perinatal factors and MIH.

Association	OR (95% CI)	*p*-Value
Maternal medication during pregnancy ↔ Premature birth	2.85 (1.28–6.37)	0.01
Low birth weight ↔ Tooth eruption disorder	3.92 (1.41–10.88)	0.009
Low birth weight ↔ Birth complications (hypoxia, respiratory distress)	5.80 (2.45–13.72)	0.0001
Fluoride application ↔ Discoloration	2.76 (1.34–5.67)	0.005
Fluoride application ↔ Caries occurrence	3.21 (1.51–6.82)	0.002
Fluoride application ↔ Hypersensitivity	2.47 (1.23–4.95)	0.01
Breastfeeding ↔ Porosity	1.34 (0.75–2.40)	0.31
Birth type ↔ Feverish illness in the first year	1.18 (0.67–2.06)	0.57
Stress ↔ Caries occurrence	1.42 (0.81–2.48)	0.23

**Table 4:** Univariate and multivariate logistic regression analysis of factors associated with hypersensitivity in children with molar–incisor hypomineralization (MIH).

Predictor	Crude OR, 95% CI (Low–Upper), *p*-Value	Adjusted OR, 95% CI (Low–Upper), *p*-Value
Maternal medication		
No	Ref	Ref
Yes	0.723 (0.179–2.924), *p* = 0.649	0.662 (0.122–3.590), *p* = 0.632
Low birth weight (<2500 g)		
No	Ref	Ref
Yes	0.584 (0.164–2.087), *p* = 0.408	0.575 (0.075–4.397), *p* = 0.594
Preterm birth (<37 weeks)		
No	Ref	Ref
Yes	0.632 (0.175–2.278), *p* = 0.483	0.461 (0.063–3.390), *p* = 0.447
Fluoride exposure (yes)		
No	Ref	Ref
Yes	0.353 (0.083–1.494), *p* = 0.157	0.251 (0.047–1.325), *p* = 0.103
Febrile illness < 1 year		
No	Ref	Ref
Yes	2.953 (0.769–11.340), *p* = 0.115	5.712 (1.011–32.271), *p* = 0.049

Legend: Ref: reference category; crude OR: crude odds ratio; adjusted OR: adjusted odds ratio; CI: confidence interval.

## Data Availability

The original contributions presented in this study are included in the article/Supplementary Materials. Further inquiries can be directed to the corresponding author(s).

## Supplementary Materials

The following supporting information can be downloaded at: https://www.mdpi.com/article/10.3390/epidemiologia7010004/s1, Table S1: Detailed Questionnaire on Developmental Enamel Defects and Perinatal Condition, Table S2: Significant and non-significant associations between perinatal factors and MIH (Chi-square test), File S1. Statistics.

## References

[1] Weerheijm K.L., Jälevik B., Alaluusua S. (2001). Molar–Incisor Hypomineralisation. Caries Res..

[2] Rodd H.D., Graham A., Tajmehr N., Timms L., Hasmun N. (2021). Molar Incisor Hypomineralisation: Current Knowledge and Practice. Int. Dent. J..

[3] Ammar N., Fresen K.F., Schwendicke F., Kühnisch J. (2025). Epidemiological Trends in Enamel Hypomineralisation and Molar-Incisor Hypomineralisation: A Systematic Review and Meta-Analysis. Clin. Oral Investig..

[4] Lopes L.B., Machado V., Mascarenhas P., Mendes J.J., Botelho J. (2021). The Prevalence of Molar-Incisor Hypomineralization: A Systematic Review and Meta-Analysis. Sci. Rep..

[5] Lygidakis N.A., Garot E., Somani C., Taylor G.D., Rouas P., Wong F.S.L. (2022). Best Clinical Practice Guidance for Clinicians Dealing with Children Presenting with Molar-Incisor-Hypomineralisation (MIH): An Updated European Academy of Paediatric Dentistry Policy Document. Eur. Arch. Paediatr. Dent..

[6] Silva M.J., Scurrah K.J., Craig J.M., Manton D.J., Kilpatrick N. (2016). Etiology of Molar Incisor Hypomineralization—A Systematic Review. Community Dent. Oral Epidemiol..

[7] Almeida L.K.Y., Carvalho T.S., Bussaneli D.G., Jeremias F. (2021). Congenital and Acquired Defects in Enamel of Primary Teeth: Prevalence, Severity and Risk Factors in Brazilian Children. Eur. Arch. Paediatr. Dent..

[8] Mlinkó É., Tábi D., Cavalcante B.G.N., Szabó B., Hegyi P., Vág J., Szabó E.V., Rózsa N.K., Varga G. (2025). Association between Systemic Exposure to Antibiotics in Early Childhood and Molar-Incisor Hypomineralization (MIH): A Systematic Review and Meta-Analysis. J. Dent..

[9] Franco M.M.P., Ribeiro C.C.C., Ladeira L.L.C., Thomaz E.B.A.F., Alves C.M.C. (2024). Pre- and Perinatal Exposures Associated with Molar Incisor Hypomineralization: Birth Cohort, Brazil. Oral Dis..

[10] Moradi H., Sheikhhassani Y., Sajadi Z., Safari M. (2025). Investigation of the Most Common Perinatal and Postnatal Risk Factors in Children with MIH Compared to Healthy (Non-MIH) Children Aged 6–12 Years in Arak City. BMC Oral Health.

[11] Wright J.T. (2023). Enamel Phenotypes: Genetic and Environmental Determinants. Genes.

[12] Costacurta M., Di Lauro M., Cornali K., Docimo R., Noce A. (2025). Developmental Defects of Enamel and Dental Caries in Pediatric Patients with Chronic Kidney Disease–Mineral Bone Disorders. Appl. Sci..

[13] Ortega-Luengo S., Feijóo-Garcia G., Miegimolle-Herrero M., Gallardo-López N.E., Caleya-Zambrano A.M. (2024). Prevalence and Clinical Presentation of Molar Incisor Hypomineralisation among a Population of Children in the Community of Madrid. BMC Oral Health.

[14] Rivera M., Karakowsky L., Medina-Solís C.E., Márquez-Corona M.d.L., Manton D.J. (2025). Prevalence, Defect Characteristics and Risk Factors Associated with Molar Incisor Hypomineralisation in Mexican Schoolchildren: A Cross-Sectional Study. Eur. Arch. Paediatr. Dent..

[15] Feltrin-Souza J., Fonseca-Souza G., Pinheiro E., Fraiz F.C., Cerri P.S. (2024). Systemic and Environmental Risk Factors Associated with Molar Incisor Hypomineralisation. Monogr. Oral Sci..

[16] Mariam S., Goyal A., Dhareula A., Gauba K., Bhatia S.K., Kapur A. (2022). A Case–Controlled Investigation of Risk Factors Associated with Molar Incisor Hypomineralization (MIH) in 8–12 Year-Old Children Living in Chandigarh, India. Eur. Arch. Paediatr. Dent..

[17] Contac L.R., Pop S.I., Voidazan S., Bica C.I. (2024). Molar Incisor Hypomineralization: Etiology, Correlation with Tooth Number Anomalies and Implications for Comprehensive Management Strategies in Children from Transylvania. Diagnostics.

[18] Juárez-López M.L.A., Salazar-Treto L.V., Hernández-Monjaraz B., Molina-Frechero N. (2023). Etiological Factors of Molar Incisor Hypomineralization: A Systematic Review and Meta-Analysis. Dent. J..

[19] Kumar A., Goyal A., Gauba K., Kapur A., Singh S.K., Mehta S.K. (2022). An Evaluation of Remineralised MIH Using CPP-ACP and Fluoride Varnish: An in-Situ and in-Vitro Study. Eur. Arch. Paediatr. Dent..

[20] Cubas Camargo R., Higashi C., Bittencourt De Abreu J.L., Hirata R. (2025). Resin Infiltration for the Esthetic Treatment of Molar-Incisor Hypomineralization: 1-Year Follow-Up. Quintessence Int..

[21] Shields S., Chen T., Crombie F., Manton D.J., Silva M. (2024). The Impact of Molar Incisor Hypomineralisation on Children and Adolescents: A Narrative Review. Healthcare.

[22] Santos P.S., Vitali F.C., Fonseca-Souza G., Maia L.C., Cardoso M., Feltrin-Souza J., Fraiz F.C. (2024). Dentin Hypersensitivity and Toothache among Patients Diagnosed with Molar-Incisor Hypomineralization: A Systematic Review and Meta-Analysis. J. Dent..

[23] Linner T., Khazaei Y., Bücher K., Pfisterer J., Hickel R., Kühnisch J. (2021). Hypersensitivity in Teeth Affected by Molar-Incisor Hypomineralization (MIH). Sci. Rep..

[24] Özgül B.M., Sakaryali D., Tirali R.E., Çehreli S.B. (2022). Does MIH Affects Preoperative and Intraoperative Hypersensitivity. J. Clin. Pediatr. Dent..

[25] Brescia A.V., Montesani L., Fusaroli D., Docimo R., Di Gennaro G. (2022). Management of Enamel Defects with Resin Infiltration Techniques: Two Years Follow Up Retrospective Study. Children.

[26] Elhennawy K., Rajjoub O., Reissmann D.R., Doueiri M.-S., Hamad R., Sierwald I., Wiedemann V., Bekes K., Jost-Brinkmann P.-G. (2022). The Association between Molar Incisor Hypomineralization and Oral Health-Related Quality of Life: A Cross-Sectional Study. Clin. Oral Investig..

[27] Enax J., Amaechi B.T., Farah R., Liu J.A., Schulze zur Wiesche E., Meyer F. (2023). Remineralization Strategies for Teeth with Molar Incisor Hypomineralization (MIH): A Literature Review. Dent. J..

[28] Alfarraj J., Alsaeed A. (2022). Clinical Management of Molar Incisor Hypomineralization Affected Molars in a Pediatric Patient Including Endodontic Treatment, Case Report and Review of the Literature. Clin. Cosmet. Investig. Dent..

[29] Patel N.S., Mehta M., Fu Y., Desai V., Lala H.S., Parikh H., Patel M.M., Thakor U.B. (2025). A Review of Early Childhood Caries: Risk Factors, Management, and Policy Recommendations. Cureus.

[30] Reissenberger T., Ebel M., Klode C., Hirsch C., Bekes K. (2022). Hypomineralized Teeth and Their Impact on Oral-Health-Related Quality of Life in Primary School Children. Int. J. Environ. Res. Public Health.

