# Supplementary material for: Perinatal Risk Factors and Clinical Correlations in Molar–Incisor Hypomineralization: A Cross-Sectional Epidemiological Study
Source: Epidemiologia (Basel). 2025 Dec 26;7(1):4. doi: 10.3390/epidemiologia7010004 (PMC12821724; doi:10.3390/epidemiologia7010004)
Supplement: Supplementary file 1 [file epidemiologia-07-00004-s001.zip › Supplementary Table S1.pdf]

Table S1. Detailed Questionnaire on Developmental Enamel Defects and Perinatal Condition

No.	Question (Item)	Response Options
1	Child's sex	Male / Female
2	Child's age	Open response (years)
3	Does the mother have any chronic or acute illness (e.g., diabetes, allergy, cardiovascular disease)?	Yes / No
4	Did the mother consume nicotine and/or alcohol during pregnancy?	Yes / No
5	Did the mother take any medication during pregnancy (e.g., antibiotics, chemotherapy, asthma or epilepsy drugs)?	Yes / No
6	Type of delivery	Vaginal / Cesarean section
7	Were any perinatal complications present (hypoxia, prematurity, respiratory distress, other)?	Yes / No / Specify
8	Child's birth weight	<2500 g / ≥2500 g
9	Did the child experience any serious illness in the first year of life (e.g., otitis media, pneumonia, gastroenteritis, UTI)?	Yes / No
10	Did the child take any medication during the first year of life (e.g., antibiotics, chemotherapy, asthma or epilepsy drugs)?	Yes / No
11	Does the child have any chronic medical condition (e.g., diabetes, allergy, epilepsy, hypocalcemia)?	Yes / No
12	Does the child have any allergies (e.g., dust, pollen, food, metals, fluoride, medication)?	Yes / No
13	If the child has a chronic condition, was it diagnosed before the age of one year?	Yes / No
14	Does the child take medication for these conditions?	Yes / No
15	Has the child ever received fluoride tablets?	Yes / No
16	Was the child breastfed?	Yes / No
17	Type of toothpaste used by the child	With fluoride / Fluoride-free / Unknown
18	Were there any problems with the eruption of primary or permanent teeth (timing, absence, abnormal shape/size)?	Yes / No
19	Age at eruption of the first permanent teeth	<6 years / 6–7 years / >7 years
20	Presence of whitish or yellowish-brownish spots on enamel	Yes / No
21	Has the child ever complained of tooth hypersensitivity (to cold, heat, or chewing, without visible caries)?	Yes / No
22	Is the enamel porous or prone to chipping?	Yes / No
23	Frequency of dental visits	>Every 6 months / Every 6 months / Once a year / >1 year
24	Frequency of toothbrushing	Once daily / Twice daily / Occasionally
25	Has the child received professional fluoride varnish treatment at the dental clinic?	Yes / No

Table S1. Detailed Questionnaire on Developmental Enamel Defects and Perinatal Condition

26	Has the child used home fluoride or fluoride-free gels for enamel strengthening?	Yes / No
27	Do the child's teeth decay easily?	Yes / No
28	Has the child ever had a permanent tooth extracted?	Yes / No
29	How would you describe your family's plastic-free lifestyle (e.g., use of BPA-free packaging)?	None / Partial (plastic reduction efforts)
30	Has the child experienced prolonged stress (e.g., family conflict, trauma, health problems)?	Yes / No
