# Supplementary material for: Perinatal Risk Factors and Clinical Correlations in Molar–Incisor Hypomineralization: A Cross-Sectional Epidemiological Study
Source: Epidemiologia (Basel). 2025 Dec 26;7(1):4. doi: 10.3390/epidemiologia7010004 (PMC12821724; doi:10.3390/epidemiologia7010004)
Supplement: Supplementary file 1 [file epidemiologia-07-00004-s001.zip › Supplementary Table S2.pdf]

Table S2. Significant and non-significant associations between perinatal factors and MIH (Chi-square test)

Association	p-value	Significance
Maternal medication during pregnancy ↔ Premature birth	0.01	Significant
Low birth weight ↔ Tooth eruption disorder	0.009	Significant
Low birth weight ↔ Birth complications (hypoxia, respiratory distress)	0.0001	Significant
Fluoride application ↔ Discoloration	0.005	Significant
Fluoride application ↔ Caries occurrence	0.002	Significant
Fluoride application ↔ Hypersensitivity	0.01	Significant
Breastfeeding ↔ Porosity	0.31	Not significant
Birth type ↔ Feverish illness in first year	0.57	Not significant
Stress ↔ Caries occurrence	0.23	Not significant
